# Band-resolved Caroli–de Gennes–Matricon states of multiple-flux-quanta vortices in a multiband superconductor

**DOI:** 10.1126/sciadv.adh9163

**Published:** 2023-09-08

**Authors:** Thomas Gozlinski, Qili Li, Rolf Heid, Ryohei Nemoto, Roland Willa, Toyo Kazu Yamada, Jörg Schmalian, Wulf Wulfhekel

**Affiliations:** ^1^ Physikalisches Institut, Karlsruhe Institute of Technology, Wolfgang-Gaede-Str.1, 76131 Karlsruhe, Germany.; ^2^ Institute for Quantum Materials and Technologies, Karlsruhe Institute of Technology, Hermann-von-Helmholtz-Platz 1, 76344 Eggenstein-Leopoldshafen, Germany.; ^3^ Department of Materials Science, Chiba University, 1-33 Yayoi-cho, Inage-ku, Chiba 263-8522, Japan.; ^4^ Institute for Theory of Condensed Matter, Karlsruhe Institute of Technology, Wolfgang-Gaede-Str.1, 76131 Karlsruhe, Germany.; ^5^ Molecular Chirality Research Centre, Chiba University, 1-33 Yayoi-cho, Inage-ku, Chiba 263-8522, Japan.

## Abstract

Superconductors are of type I or II depending on whether they form an Abrikosov vortex lattice. Although bulk lead (Pb) is classified as a prototypical type-I superconductor, we show that its two-band superconductivity allows for single-flux-quantum and multiple-flux-quanta vortices in the intermediate state at millikelvin temperature. Using scanning tunneling microscopy, the winding number of individual vortices is determined from the real space wave function of its Caroli–de Gennes–Matricon bound states. This generalizes the topological index theorem put forward by Volovik for isotropic electronic states to realistic electronic structures. In addition, the bound states due to the two superconducting bands of Pb can be separately detected and the two gaps close independently inside vortices. This yields strong evidence for a low interband coupling.

## Teaser

Microscopic evidence reveals a multiple-flux-quanta vortex phase in bulk lead.

## INTRODUCTION

The classical solutions in the Ginzburg-Landau theory allow a thermodynamic classification of superconductors into type I and type II. Decisive for their behavior in magnetic field is the interface energy between the superconducting and normal phase driven by the ratio of the London magnetic penetration depth λ_L_ and the superconducting coherence length ξ. For Ginzburg-Landau parameters κ = λ_L_/ξ < 1/√2, type-I behavior and for >1/√2, type-II behavior was predicted (*1*). A type-I superconductor is characterized by a positive interface energy and an attractive vortex-vortex interaction favoring an intermediate state with large normal domains (*2*). A type-II superconductor is characterized by a negative interface energy and a repulsive vortex-vortex interaction that favors an Abrikosov lattice of single-flux-quantum vortices in the mixed phase (*3*).

Flux quantization Φ = *m*Φ_0_ in units of the flux quantum Φ_0_ = *h*/2*e* is one of the most fundamental traits of the underlying off-diagonal long-range order of the superconducting condensate (*4*). *m* is the integer winding number of the vortex. This number of confined flux quanta is expected to affect the size and shape of the vortex. More quantitatively, Volovik (*5*) demonstrated for superconductors with isotropic Fermi surfaces that one can determine *m* from an index theorem, provided one can measure the wave function of the quasiparticle bound states that form in the vortex core. The vorticity is particularly ambiguous, and hence interesting near the Bogomol’nyi point, κ = 1/√2. Then, vortices near *T*_c_ behave like noninteracting particles and the vortex configuration is infinitely degenerate (*6*–*9*) such that multiple-flux-quanta (or giant) vortices may be stabilized (*10*). This degeneracy is lifted below *T*_c_ and a transitional region, in which the superconductor cannot be categorized into either of the types described above, emerges (*11*). When leaving the Ginzburg-Landau limit toward lower temperatures and especially, when considering multiple-band (multiband) superconductors, microscopic interactions are predicted to become increasingly important: The vortex-vortex interaction energy can become nonmonotonic in distance through the existence of multiple, distinct superconducting coherence lengths ξ_i_ (*12*, *13*) and topological hysteresis due to transitions between flux tubes and laminar pattern influences the flux patterns of the intermediate state (*14*).

In the past, the crucial parameter κ has been tuned toward the type-II regime by either an increase of λ_L_ or reduction of the effective ξ, i.e., by using thin films below a critical thickness (*15*, *16*), by incorporation of impurities [for relevant experiments see (*11*) and references within] or by interface scattering, e.g., Pb/Si(111) (*17*) or Pb on black phosphorus (*18*). This approach, however, has the drawback that the quasiparticle bound states in the vortex core are considerably smeared out such that the index theorem cannot be applied (*17*–*22*).

Here, we take the alternative approach and study the two-band superconductor Pb in the clean limit in form of a bulk single crystal Pb(111) at 45 mK. In this respect, the multiband superconductor Pb, which is closest to the Bogomol’nyi point of all elemental superconductors, is a good candidate to study the transitional phase at temperatures well below *T*_c_. We map the quasiparticle bound states for the two superconducting gaps of Pb with high energy and spatial resolution and use the index theorem including realistic band structures to determine the winding number of single- and multiple-flux-quanta vortices. Ultimately, we show that our observations allow to investigate the interband coupling of the two gaps.

## RESULTS

### Superconducting gaps and intermediate state

**Fig. 1.**
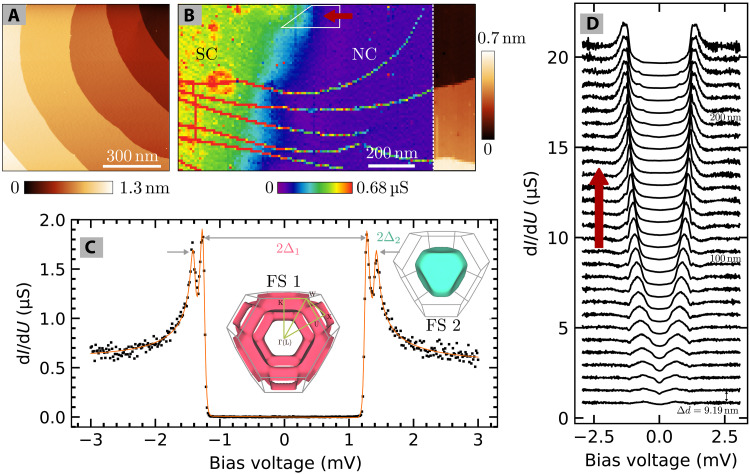
Superconducting properties and intermediate state. (**A**) Topographic scan image of the Pb(111) surface after the cleaning procedure. (**B**) d*I*/d*U* map at *U*_t_ = 1.3 mVshowing a typical domain wall in the intermediate state at *B* = 23 mT. Right overlay: Continuation of the map showing the corresponding topography. (**C**) Differential conductance in the superconducting state (or superconducting domain) (black points) including a two-gap BCS fit (orange) with ∆_1_ being the smaller and ∆_2_ being the larger gap. Inset: 3D models of Fermi surface sheets 1 and 2 [from (*64*)] responsible for superconducting gaps ∆_1_ and ∆_2_ respectively. (**D**) d*I*/d*U* spectra along a cross section from normal conducting to superconducting domain. The area is marked in white in (B) and the profile direction is indicated by a red arrow. Individual spectra are offset with respect to each other by 0.7 μS. The spectra were locally averaged over a straight part of the domain boundary and recorded in distance increments of ∆*d* = 9.19 nm.

After several cycles of sputtering and annealing, we obtained a clean Pb(111) surface with wide terraces and monoatomic steps, as shown in Fig. 1A. Upon zero-field cooling the Pb(111) sample to 45 mK it enters its superconducting state below *T*_c_ ≈ 7.2 K (*23*). Because of our low electronic temperature of less than 100 mK (*24*), we are able to resolve the two gaps (*25*) in the density of states by scanning tunneling spectroscopy, even with a normal conducting tip. Figure 1C shows the differential conductance (d*I*/d*U*) in the superconducting state as black dots, including a temperature broadened two-gap fit to the density of states in the Bardeen-Cooper-Schrieffer (BCS) theory in orange. We determine the superconducting gaps to be ∆_1_ = (1.26 ± 0.02) meV (smaller gap) and ∆_2_ = (1.40 ± 0.02) meV (larger gap) in good agreement with previous measurement of the difference of the two gaps (*25*). The electronic temperature of the fitted curve is *T* = (121 ± 1) mK. The intensity difference of the two coherence peaks has previously been attributed to the *k*-dependent tunneling matrix elements and the larger gap has been assigned to the tubular Fermi surface sheet (*25*). This is in contrast to Bogoliubov–de Gennes–based Korringa-Kohn-Rostoker calculations (*26*), which deduced an opposite band-to-gap assignment. As will be discussed below, our study of the quasiparticle bound states in the vortices confirms the band-to-gap assignment of Saunderson *et al.* (*26*). We will from here on index the bands and Fermi surfaces according to their superconducting gap, i.e., the tubular Fermi surface responsible for ∆_1_ as Fermi surface 1 (FS 1) and the compact Fermi surface responsible for ∆_2_ as Fermi surface 2 (FS 2) (Fig. 1C).

After applying a perpendicular magnetic field of *B* = 85 mT, which is above the critical field μ_0_*H*_c_ ∼ 80 mT (*27*), magnetic flux enters the sample from the sides and completely destroys superconductivity. Upon decreasing the field again below *H*_c_, the Landau intermediate state is reached. It is detected by recording the differential conductance at the coherence peak of ∆_2_ while ramping the field down. Once a jump to the superconducting state below the tip is detected, the ramp is stopped. This ensures that one typically finds both, superconducting and normal conducting, areas in the scan range of the scanning tunneling microscopy (STM) setup of 1.4 × 1.4 μm^2^. The intermediate state is characterized by large normal and superconducting domains. The shapes and sizes of these domains in the intermediate state of lead have been extensively studied by magneto-optical methods revealing the strong dependencies on temperature, sample shape, and magnetic protocol (*14*, *28*–*30*).

A typical domain wall in the intermediate state is shown in the d*I*/d*U* map in Fig. 1B. At a tunneling bias of *U*_t_ = 1.3 mV, the normal conducting domains show up as areas of low conductance (purple) and the superconducting domains as areas of high conductance (green/yellow). Note that atomic step edges of the surface cause a contrast in d*I*/d*U* (here, visible as yellow-red lines) as typically found in STM experiments and is illustrated by the overlay on the right, where we switch from d*I*/d*U* to topographic map at the white dotted line. The contrast at step edges comes from a combination of the Smoluchowski effect, locally reducing the work function at step edges, and technical aspects of scanning like a finite speed of the feedback mechanism and the tip geometry at the atomic scale. For our purpose, these lines can be seen as an artifact. A cross-sectional line scan across the domain wall, as in Fig. 1D, shows how both gaps change from zero to their maximum on the length scale of the coherence length. The local recovery of superconductivity agrees well with reported coherence lengths of ξ = 87 nm (*23*). For a detailed analysis of the coherence length ξ_1,2_ of the two bands measured inside vortices, we refer to the next section.

### Single-flux-quantum vortices

Inside the normal-core region of vortices, electronic bound states that lie within the superconducting gap are localized. These in-gap states were first theoretically studied by Caroli, de Gennes, and Matricon (CdGM) in 1964 (*31*). The CdGM states for isotropic bands can be characterized by their orbital angular momentum number μ and their energy *E*. The energy spacing of the discrete CdGM states is of the order of ∆^2^/*E*_F_, where *E*_F_ is the Fermi energy, and the discrete states thus form a quasicontinuum or branch of CdGM states for most superconducting materials. The CdGM states with low μ are confined closer to the vortex core than the ones with high μ, thus leading to a one-to-one correspondence between the angular momentum and the real-space behavior of the bound-state wave function. The higher the angular momentum μ or energy *E*(μ), the further away from the vortex center are the states localized (*32*). In case of an *m*-flux quanta vortex, *m* individual CdGM branches exist (*5*, *33*–*36*), leading to a topological index theorem: The number of zero-energy crossings of CdGM state branches with varying angular momentum is directly related to the vorticity (*5*, *33*, *34*). This theorem translates to real space and the number of zero-energy crossings of CdGM state branches with varying radius from the vortex core is related to the vorticity.

STM allows to measure the variation of the local density of states (LDOS) inside the vortex and thus to determine the winding number of the vortex using the index theorem. In 1989, Hess *et al.* (*37*) experimentally confirmed that CdGM states exist in single-flux-quantum vortices by STM, but for vortices with multiple flux quanta, although studied in thin films with electron holography (*38*) and scanning Hall probe microscopy (*39*), their predicted bound states still lack experimental verification (*35*, *36*, *40*, *41*).

**Fig. 2.**
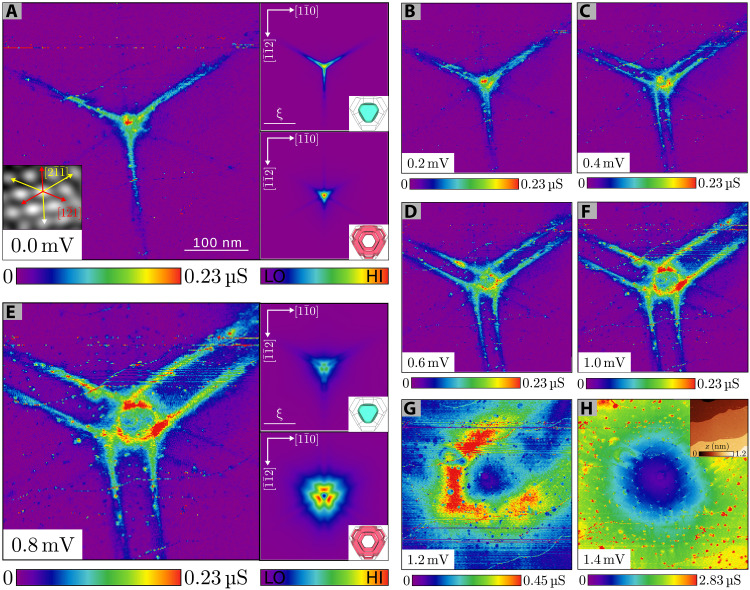
Single-flux-quantum vortex signature. (**A** to **H**) d*I*/d*U* maps (*B* = 0 mT) of a single-flux-quantum vortex at different bias voltages *U*_t_ (indicated in the bottom left corner of each image) displaying the quasiparticle density at the surface. Right panels in (A) and (E) show the simulated LDOS of the bands 1 and 2 for the respective energy. The inset in (A) shows a 2D fast Fourier transform–filtered topographic image of the Pb(111) lattice. Bulk crystal directions (red/yellow arrows) have been determined from glide planes. The inset in (H) shows the topographic image of the same area recorded at *U*_t_ = 1.4 mV.

Using the detection method described in the previous section, we are also able to find isolated, round normal conducting domains (appearance at *eU* = ∆_2_) of ≈100 nm in diameter. An example is shown in Fig. 2H. As will be shown later, these are vortices in the superconductor with integer number of flux quanta. They appear alongside the normal intermediate state domain structure discussed in the previous section and without an ordered vortex pattern, as would be the case for a type-II superconductor (see the Supplementary Materials). The finding of such small normal conducting domains is unexpected considering that the domain wall energy in type-I superconductors is positive and the system thus tries to maximize its domain size. Even more unexpected is the fact that we found this shape after ramping the field down from the critical field. Magneto-optical measurements of cylindrical-shaped intermediate state lead samples at 4.5 K reveal that normal domains are only tubular upon increasing magnetic field; after ramping down from the critical field, the preferred structure is laminar (*28*). A deciding factor for the intermediate state domain structure on a microscopic scale could be the effect of flux branching (*42*–*44*). Since the overall domain structure in our experiment, however, consists of domains of various shapes and sizes, we argue that this finding is only consistent with circumstances under which vortices interact weakly and have a nonmonotonous interaction energy causing hysteresis, i.e., a superconductor in the transitional phase (close to the Bogomol’nyi point) at temperatures well below *T*_c_. The possibility of vortex pinning at large surface defects can be discarded as the topographic image of the vortices only shows the atomically flat Pb(111) surface, as shown in the inset of Fig. 2H. The role of pinning of vortices at invisible bulk defects below the surface remains unclear. Repeating our magnetic protocol several times, vortices may appear at similar positions, which can, however, be interpreted as consequence of the protocol, i.e., stopping the magnetic ramp, when a vortex is under the tip. We also find the vortices to be mobile when varying the magnetic field (see next section).

To determine the amount of flux carried by the small normal domains, we record differential conductance (d*I*/d*U*) maps at subgap energies, which essentially show the LDOS of the CdGM states. At zero bias voltage, we find a threefold symmetric state in form of a star with a maximum in the star’s center (Fig. 2A) inside the normal domain similar to star-shaped CdGM states seen by Hess *et al.* (*45*) in 2H-NbSe_2_. The quasiparticle density of states stretches over 100 nm in the ⟨$2\bar{1}\bar{1}$⟩ directions (blue/green). In addition, three weak arms (dark blue) along the ⟨$12\bar{1}$⟩ directions are visible. With increasing energy (independent of sign of bias voltage), the star’s arms split into two with increasing splitting distance, while the central peak splits nearly isotropically to a ring shape (Fig. 2, B to F). For *E* ≤ ∆_1_, the strong arms are still visible and the ring reaches its maximal size (Fig. 2G). For *E* ∼ ∆_2_, the vortex shows as a relatively round area of low conductance with ≈100 nm in diameter (Fig. 2H).

For bands with anisotropic Fermi velocity, like in our case, the index theorem is not straightforward applicable since the radial symmetry is removed and a radial-dependent measurement does not ensure crossing all diabolical points (*5*, *33*). Diabolical points here mean that the points where the semiclassical particle and hole spectrum meet. The degenerate gapless fermionic excitations or “zero modes” at these points carry the topological charge (singularity in the phase) and owe their name to the double-conical (diabolo) dispersion in parameter space (*46*). Here, a realistic band structure needs to be considered. We, thus, simulated the quasiparticle trajectories inside a vortex carrying one flux quantum within the quasiclassical Eilenberger theory, including the Fermi velocity of each band obtained from density functional theory (DFT) calculations and compare the obtained LDOS maps to our experimental results (for details, see Materials and Methods). Right panels in Fig. 2 (A and E) display the solutions reproducing the star shape for the compact Fermi surface 2 and the ring-like structure of the tubular Fermi surface 1. The simulations confirm that the observed states are the signature of a vortex in Pb(111) containing a single flux quantum. The ring-shaped states, related to the tubular Fermi surface (band 1) of nearly isotropic Fermi velocity, show the expected behavior. At *U* = 0, it creates a sharp maximum in the center of the vortex that splits into a ring of increasing diameter upon variation of the voltage away from *U* = 0. For the compact Fermi surface (band 2) with large flat structures in the Fermi surface and anisotropic Fermi velocity, a star-shaped structure is predicted whose arms split into two when going away from *U* = 0. The observation of two sets of CdGM states in the experiment is in good agreement with the semi-classical treatment of separate Fermi surfaces, which already indicates that the coupling between the two Fermi surfaces is weak, i.e., the rate of scattering between the two Fermi surfaces is lower than the inverse time for the electron round trip around the vortex.

Star-shaped CdGM states have first been found by Hess *et al.* (*45*) in 2H-NbSe_2_, which due to the crystal symmetry have sixfold rotational symmetry. Similarly, CdGM states of vortices in Nb(110) were shown to exhibit twofold rotational symmetry (*47*). Pb crystallizes in the face-centered cubic structure and belongs to the point group $\mathrm{Fm}\bar{3}m$. The electronic structure therefore carries a threefold rotational symmetry about the [111] axis. Because of the discrete rotational symmetry, the angular momentum is not a good quantum number and the states mix. In general, the mixing results in states with different lateral confinement for different angles. At zero bias, the low angular momentum states carry the largest weight and lead to a maximum in the center of the vortex. At higher bias, states of larger angular momentum become increasingly important, leading to a movement of the maxima away from the center, i.e., the star splits. The large variation in quasiparticle localization depending on the angle from ∼10 nm to over 100 nm agrees with the strong anisotropy of the three-dimensional (3D) Fermi velocity in the compact band. The extension of the CdGM states beyond the vortex core illustrates that the sample is in the clean limit.

**Fig. 3.**
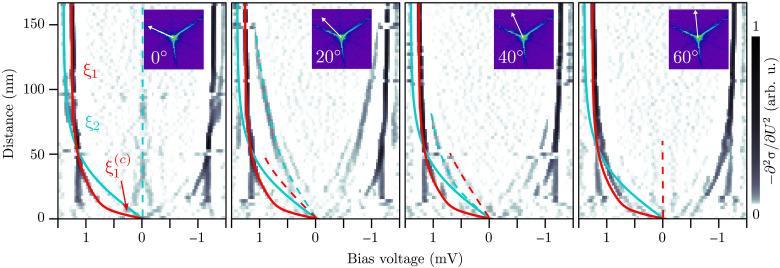
Coherence lengths in the normal single-flux-quantum vortex. Angle-dependent radial measurement of the vortex core states from the vortex in Fig. 2. Displayed is the second derivative of the differential conductance −∂^2^σ/∂*U*^2^ to highlight maxima in the LDOS. The point distance of single spectra is ∆*d* = 2.57 nm. Marked are the opening of ∆_1_ (red line), the opening of ∆_2_ (cyan line), and the CdGM states of band 1/2 (red/cyan dashed line). The inset shows the direction of the *y* axis.

Star-shaped vortices with long arms have been recently observed in the Abrikosov lattice of La(0001) (*48*), owing to the large anisotropy of the responsible band’s in-plane Fermi velocity. In Pb with its two bands, one may expect that the individual gaps will close independently when approaching the vortex center. Figure 3 displays the evolution of the gaps and in-gap states in the second derivative of d*I*/d*U* with respect to *U*, as function of the distance from the vortex center and direction (indicated in the inset). The energy of the coherence peaks appear as dark lines and energy of the CdGM states as faint dark lines. To clarify, we plot gap evolution functions ∆(*r*) = ∆_∞_ tanh(*r*/ξ) for band 1 (red line) and band 2 (cyan line), which we fitted to the coherence peaks in the leftmost panel. In the case of band 1, we find that the gap opening can only be described when considering a nonuniversal ξ, i.e., ξ_1_ for the behavior far from the core and ξ_1_ for the behavior deep in the core of the vortex. Dashed lines in red [cyan] are guides to the eye for the spatially anisotropic CdGM states of band 1 [2], i.e. the faint star arms in the ⟨$12\bar{1}$⟩ [pronounced star arms in the ⟨$2\bar{1}\bar{1}$⟩] directions. For large distances from the center, the gaps ∆_1_ and ∆_2_ decrease toward the center in parallel with roughly the same length scale ξ_1_ ∼ ξ_2_ ∼ 45 nm. In the whole range, ∆_2_ follows a simple tanh function. At 50 nm, however, ∆_1_ crosses ∆_2_ and stays larger than ∆_2_. Thus, for the band 1, the gap size deviates from the tanh function near the center and decreases on a shorter length scale. This observation for a single-band superconductor is known as the Kramer-Pesch effect (*49*). It was quantified by numerical calculations by Gygi and Schlüter (*32*) for the vortex core size of type-II superconductors. The slope of ∆_1_ near the core corresponds to a core size $\xi_{1}^{\left(c\right)}={\text{Δ}}_{1}(\infty ){\left[\lim_{r\to 0}\frac{d{\text{Δ}}_{1}(r)}{dr}\right]}^{-1}$ of only ∼ 10 nm. In our self-consistent calculation of the pair-potential ∆(*r*) for an isotropic vortex in the quasiclassical theory (see the Supplementary Materials), this Kramer-Pesch shrinking effect is also present and leads to substantial deviation from a tanh function with one universal ξ. Note that theory in the clean limit, however, predicts a core shrinking proportional to *T*/*T*_c_ when lowering the temperature. At very low temperatures *T* ≪ *T*_c_ the slope of the order parameter d∆/d*r* at the vortex center is even predicted to become infinite, which would show as a jump of ∆(*r*) at *r* = 0 that is smoothed out over the distance ξ*T/T*_c_ (*33*). Experimentally, we do not find this extreme shrinking. Meanwhile, the “squeezing” of low angular momentum states in the vortex core and thus the Kramer-Pesch effect is absent for the star, i.e., CdGM states of band 2. Consequently, $\xi_{1}^{\left(c\right)}$ deviates from $\xi_{2}^{\left(c\right)}$, which indicates that intraband coupling dominates over interband coupling, i.e., the two bands are sufficiently decoupled from each other, despite the gap sizes being not too different (*50*). For an isotropic two-band superconductor, the shrinking of the core region for only one of the bands has also been predicted in the case of rather weakly coupled bands (*51*). In this theoretical prediction, the Kramer-Pesch effect for the two weakly coupled bands depends on the ratio of the Fermi velocities. The band with the larger Fermi velocity is expected to show a smaller Kramer-Pesch effect. This fully agrees with our observation of a lower Kramer-Pesch effect for band 2, which has a larger Fermi velocity according to our calculations and the band calculations by Saunderson *et al.* (*26*). Again, this hints toward a low interband coupling in Pb.

### Anomalous single-flux-quantum vortices

**Fig. 4.**
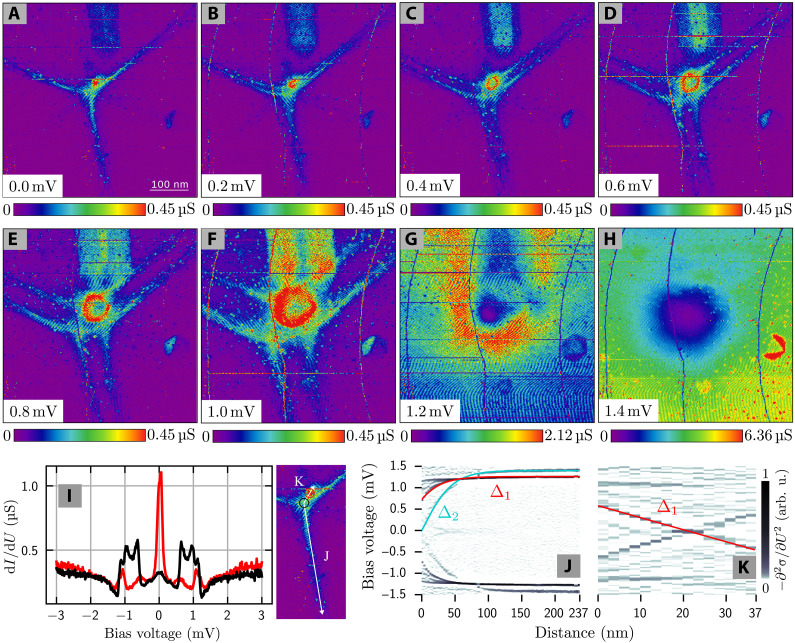
Anomalous vortex signature. (**A** to **H**) d*I*/d*U* maps (*B* = 19 mT) at different bias voltage for an anomalous vortex (compared to the normal vortex configuration from Fig. 2). (**I**) Right: Enlarged zero bias d*I*/d*U* map of the anomalous vortex including the position of line spectra (white arrows) and single spectra locations (red/black circle). Left: Single d*I*/d*U* spectra in the star center (black) and ring center (red) revealing zero bias peaks of different amplitude. (**J** and **K**) Heatmaps of the second derivative of the differential conductance −∂^2^σ/∂*U*^2^ along the cross sections marked in (I). Red and cyan lines follow ∆_1_ and ∆_2_, respectively.

Besides the regular vortex situation, we find vortices, in which the two sets of CdGM states are laterally displaced. Figure 4 shows such an anomalous vortex. Both the ring center and the star center of the CdGM states are independently movable by a change in magnetic field and their relative displacement can be manipulated into different configurations, even back to the normal configuration from Fig. 2 (see the Supplementary Materials). We explain this by two effects.

First, a change in magnetic field laterally moves the vortex and with it, the two sets of CdGM states. Second, a change in magnetic field can lead to a tilting or bending of the flux lines away from a normal direction to the surface. This leads to a breaking of the cylinder symmetry and can displace the two sets of CdGM states relative to each other. The individual sets of CdGM states behave as those of the regular vortices, except for their relative displacement (see Fig. 4, A to H).

The lateral displacement allows an independent probing of the state sets and thus, an independent identification of their bands. By looking at single bias spectra at the star center (black) and the ring center (red) in Fig. 4I, we find that the amplitude of the peak at zero bias is three times larger for the ring than for the star, which is supported by our separate band simulations from earlier and can be explained by the larger lateral confinement of low angular momentum states in band 1 compared to band 2. Figure 4 (J and K) reveals maxima in the LDOS along the cross sections marked in the right panel of Fig. 4I in the form of heatmaps of the differential conductance’s second derivative with respect to bias voltage. It becomes apparent that ∆_2_ (cyan line) closes entirely, whereas ∆_1_ (red line) does not completely close in the star’s center (black circle). Instead, ∆_1_ closes about 20 nm away from the star center (red circle). Consequently, the superconducting gap ∆_1_ is linked to the ring and ∆_2_ to the star. The fact that the quasiparticles of ∆_1_ and ∆_2_ can be independently displaced with respect to each other rules out rigid interband coupling.

### Multiple-flux-quanta vortices

**Fig. 5.**
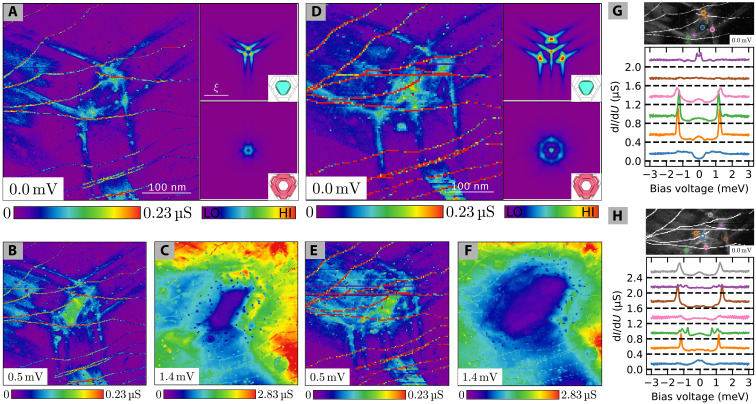
Multiple-flux-quanta vortex signature. (**A** to **F**) d*I*/d*U* maps of an *m* = 2 vortex at *B* = 0 mT (A to C) and an *m* = 3 vortex at *B* = 33 mT [(D) to (F)] at selected bias voltages. Right panels in (A) and (D) show the simulated LDOS of the bands 1 and 2 for the respective *m* quanta vortex and energy. (**G** and **H**) Bias spectroscopies (bottom) recorded with the tip at specific locations marked in the zero bias maps (top) of the *m* = 2 (G) and *m* = 3 (H) vortex. Individual spectra are offset by 0.4 μS for clarity. Black dashed lines indicate their respective zero conductance baselines.

We also observed larger vortices in the experiments. Figure 5 displays two examples. Figure 5 (A to C) shows a vortex with two flux quanta. Our semiclassical calculations indicate that for each flux quantum and each band, a branch of CdGM states is present. As a result, for the vortex with two flux quanta, the band 2 causes a structure with two arms per direction at zero bias (see Fig. 5A) that individually split into two arms with bias voltage (see Fig. 5B) just as in the single-flux-quantum vortex. An analogous behavior is observed in the vortex with three flux quanta shown in Fig. 5 (D to F). Both vortices are larger than the single-flux-quantum vortex (compare Fig. 2H with Fig. 5, C and F). Further, they deviate from a round shape and laterally grow with the number of flux quanta. The number of flux quanta in the vortices does not seem to be limited. We observed giant vortices with over 10 flux quanta (see the Supplementary Materials). For band 1, the problem of multiple-flux-quanta vortices is similar to that of a single-band superconductor with a spherical Fermi surface and has been studied in detail by Volovik (*5*, *33*). In essence, an *m*-flux quanta vortex results in *m* branches of circular CdGM states of different radii. Because of symmetry, at zero bias and for odd *m*, a central spot is formed by one branch of the CdGM states and the other states form pairs of increasing ring diameters. The CdGM states at energies away from the Fermi level evolve by changing the radii. Thus, the central CdGM state turns from a spot of zero radius to a ring of finite radius, and the pairs of CdGM states split in their radius. For an even *m*, no central spot is present at the Fermi energy but only pairs of CdGM states with distinct radii exist that again split when moving away from the Fermi energy. This is the essence of the topological index number theory of Volovik. In our case, we find a central spot of high differential conductance at zero bias voltage for the vortices with *m* = 1 and *m* = 3, while there is no central spot for the vortex with *m* = 2 in agreement with the topological index number theory. When going away from the Fermi energy, the ring-shaped and spot-like CdGM states overlap with the star-shaped states such that their distinction becomes impractical. Tunneling spectra at selected locations inside the vortex showing the very same effects are shown in Fig. 5 (G and H).

While the original formulation of the Volovik’s index number theorem is not directly applicable in real space (that is, counting the number of zero modes in the vortex core as function of radius), in our case of an anisotropic Fermi surface, we still believe that the number of parallel arms of our star-shaped CdGM state pattern at zero energy exactly reflects the winding number *m* of the vortex. The reason for our confidence lies in the fact that the diabolical points in *k* space, that need to be counted, still lie at zero energy, it is just not obvious, which path in real space crosses each of them exactly once. Comparing the axisymmetric problem with the problem at hand, it becomes obvious that a parametrization in terms of the set of quantum numbers (*k_r_*, *k*_ϕ_, and *k_z_*) has to be replaced by an irreducible representation of the crystallographic point group. The flat parts of Fermi surface 2 focus the quasiparticles into high-symmetry directions and instead of a localization of different CdGM state branches at different radii, they now localize at different impact parameters.

In total, we studied 99 vortices below 40 mK, which all show the behavior presented here. At last, we tested whether vortices would also be present at temperatures much closer to *T*_c_. Our measurements at 4.3 K revealed that it is substantially harder to trap a vortex in our scan frame, but we managed to in two cases (see the Supplementary Materials).

## DISCUSSION

We report the observation of single-flux-quantum and multiple-flux-quanta vortices in a traditional type-I bulk superconductor, i.e., single-crystal Pb(111), by low-temperature STM. We also demonstrate a robust determination method for the winding number of the vortex by usage of a topological index theorem, which relates the number of flux quanta and CdGM state branches. The spatial anisotropy of the quasiparticle states inside the vortex reflects the crystal symmetry, and its shape is governed by the anisotropic Fermi velocity in the superconducting bands. An influence of neighboring flux lines can be ruled out due to the absence of an ordered flux pattern. In addition, we could show how CdGM states from two weakly coupled bands interact with each other in a flux line. While the generically unavoidable interband coupling leads to the formation of a single coherence length and order parameter right at *T*_c_ (*52*), the physics below *T*_c_ is considerably richer, in particular in the regime of weak interband coupling (*50*, *51*). Of particular interest is the emergence of mixed gap modes (*51*) or the possibility of the observation of the rather elusive Leggett mode in the limit of weak interband coupling (*53*), i.e., a collective fluctuation of the relative phase of the two bands, for superconducting Pb. We experimentally demonstrate that for systems with multiband pairing, the transitional region of the hysteretic behavior expands due to microscopic interactions and also allows for interesting vortex configurations, like vortex clusters or multiple-flux-quanta vortices (*54*). The emergence of such vortices in the intermediate state of a prototypical type-I superconductor is very unexpected and demonstrates that the low-temperature flux patterns of two-band superconductors rank on a spectrum between types I and II. Not only does the existence of vortices in Pb present an alternative test ground of vortex physics in multicomponent superconducting systems, the variability of the vortex winding number also suggests a combination with topological crystal defects that was recently predicted to result in topological quasiparticle states, like Majorana zero modes, under certain circumstances (*55*, *56*).

## MATERIALS AND METHODS

### Experimental details

The experiments were performed with a home-built STM with dilution refrigeration, which can reach a base temperature of 25 mK in a magnetic field of up to 7.5 T (*24*). In our setup, the bias voltage *U*_t_ is applied between sample and common machine ground so that a positive bias voltage probes the unoccupied states of the sample. The STM chamber is kept at a base pressure of 1 × 10^−10^ mbar. The single-crystal Pb(111) (miscut angle, ±0.1°; purity, 99.999%) has been purchased from MaTecK GmbH. It has cylindrical (hat) shape with a diameter of 8 mm and a thickness of 2 mm. At a base pressure of 1 × 10^−10^ mbar, the Pb crystal was prepared in cycles of sputtering with Ar^+^ ions of 3 keV and subsequent annealing at 190°C and directly transferred into the STM in situ. A tungsten tip was prepared by high-temperature flashing and soft dipping into a Au(111) surface to avoid picking up Pb atoms. The measurements (except for the one at 4.3 K) were all performed below 45 mK. After zero-field cooling the Pb crystal, the vortices were formed by ramping the perpendicular magnetic field from 0 to 85 mT and back down to a constant value below the critical field. Note that 80 mT is the critical magnetic field for Pb (*27*). The ramping rate was 5 mT/min.

The differential conductance was measured using a lock-in amplifier at a frequency of 3.4 to 3.6 kHz and AC peak amplitude $U_{\mathrm{ac}}^{\left(\mathrm{PK}\right)}$ between 10 and 100 μV (for details, see the Supplementary Materials). d*I*/d*U* maps at subgap energies were recorded in a multipass configuration: In the “record” phase the tip records the *z*-profile at the feedback condition (constant tunneling current of *I*_t_ at *eU*_t_ > ∆) and in the “play” phase the *z*-profile is repeated at a different bias voltage. To increase the signal for subgap energies an offset is often added to the *z*-profile bringing the tip closer to the surface. This record and play phase alternation is performed line for line until the entire area has been scanned.

### Calculation details

To obtain the simulated LDOS for vortices containing an arbitrary number of flux quanta, we used the Riccati parametrization of the quasiclassical 3D Eilenberger equations as proposed in (*57*) and numerically solved the 1D differential equations under appropriate boundary conditions (for details, see the Supplementary Materials) for each band separately. Motivated by our experimental findings, we assumed a radial symmetric local pair potential ∆(*r*) in the plane perpendicular to a vortex line with *s*-wave symmetry that vanishes in the vortex center. We used the ratio ∆_2_(∞)/∆_1_(∞) obtained from the experiment. We respected the broken translation symmetry at the crystal surface by a work function term. The magnetic vector potential was set to zero for all calculations shown in the main text. An inclusion of a magnetic vector potential of appropriate form only yielded small quantitative deviations from the zero-field case (see the Supplementary Materials), yet drastically increased the required computation time, which is why we refrained from it for the LDOS maps.

Density functional calculations of the electronic structure of Pb were carried out in the framework of the mixed-basis pseudopotential method (*58*, *59*). The electron-ion interaction was represented by norm-conserving relativistic pseudopotentials (*60*). Spin-orbit coupling was incorporated within the pseudopotential scheme via the Kleinman’s formulation (*61*) and was consistently taken into account in the charge self-consistency cycle using a spinor representation of the wave functions. Further details of the spin-orbit coupling implementation within the mixed-basis pseudopotential method can be found in a previous publication (*62*). For higher accuracy, 5*d* semicore states were included in the valence space. The deep *d* potential is efficiently treated by the mixed-basis approach, where valence states are expanded in a combination of plane waves and local functions. Here, local functions of *d* type at the Pb sites were combined with plane waves up to a kinetic energy of 20 Ry. Brillouin zone integration was performed by sampling a 32 × 32 × 32 *k*-point mesh (corresponding to 2992 *k-*points in the irreducible part of the Brillouin zone) in conjunction with a Gaussian broadening of 0.2 eV. The exchange-correlation functional was represented by the local density approximation in the parameterization of Perdew and Wang (*63*).

This DFT technique was applied to obtain Fermi surface properties entering the Eilenberger equations. Band energies were calculated on fine radial grids for a cylindrical coordinate system taking the [111] direction as the *z* axis, to determine Fermi momenta *k*_F_ for each of the two relevant bands. At each *k*_F_, 3D Fermi velocities *v*_F_ were then calculated taking numerical derivatives of band energies around this point. The optimized lattice parameter *a* = 4.89 Å was used throughout.

## Supplementary Materials

### This PDF file includes:

Extended Methods Supplementary Notes 1 to 5 Supporting Data 1 and 2 Table S1 Figs. S1 to S10 Legends for data S1 to S6 References
