# Supplementary material for: Band-resolved Caroli–de Gennes–Matricon states of multiple-flux-quanta vortices in a multiband superconductor
Source: Sci Adv. 2023 Sep 8;9(36):eadh9163. doi: 10.1126/sciadv.adh9163 (PMC10491140; doi:10.1126/sciadv.adh9163)
Supplement: Supplementary file 1 — Extended Methods Supplementary Notes 1 to 5 Supporting Data 1 and 2 Table S1 Figs. S1 to S10 Legends for data S1 to S6 References [file sciadv.adh9163_sm.pdf]

Science Advances
AAAS

Supplementary Materials for

Band-resolved Caroli–de Gennes–Matricon states of multiple-flux-quanta vortices in a multiband superconductor

Thomas Gozlinski *et al.*

Corresponding author: Thomas Gozlinski, thomas.gozlinski@kit.edu

Sci. Adv. **9**, eadh9163 (2023)
DOI: 10.1126/sciadv.adh9163

The PDF file includes:

Extended Methods
Supplementary Notes 1 to 5
Supporting Data 1 and 2
Table S1
Figs. S1 to S10
Legends for data S1 to S6
References

Other Supplementary Material for this manuscript includes the following:

Data S1 to S6

Contents

Extended Methods

Supplementary Note 1: Anomalous vortices in varying magnetic fields

Supplementary Note 2: Giant vortex

Supplementary Note 3: Particle-hole and time-reversal symmetry

Supplementary Note 4: Vortex at 4.3 K

Supplementary Note 5: Self-consistent calculation of Δ

Supporting Data 1: Absence of ordered vortex pattern

Supporting Data 2: dI/dU data for Fig. 3 and 4

Extended methods

STM measurement conditions : Tab. S1 summarizes the most important STM/STS measurement parameters used to obtain the figures in the main text and supplementary material.

3D Eilenberger calculations : We follow Ref. (*57*) in notation and describe how we solved the quasiclassical Eilenberger equations (*65, 66*)

$$-\hbar \boldsymbol{v}_F \nabla \hat{g}(\boldsymbol{r};\boldsymbol{p}_F,i\epsilon_n) = \left[\begin{pmatrix} i\epsilon_n + \boldsymbol{v}_F e\boldsymbol{A}(\boldsymbol{r}) & -\Delta(\boldsymbol{r},\boldsymbol{p}_F) \\ \Delta^\dagger(\boldsymbol{r},\boldsymbol{p}_F) & -i\epsilon_n - \boldsymbol{v}_F e\boldsymbol{A}(\boldsymbol{r}) \end{pmatrix}, \hat{g}(\boldsymbol{r};\boldsymbol{p}_F,i\epsilon_n) \right], \quad (1)$$

that hold for $k_F\xi \gg 1$. The quasiclassical Green's function propagator

$$\hat{g} = \begin{pmatrix} g(\boldsymbol{r},\boldsymbol{p}_F,i\epsilon_n) & f(\boldsymbol{r},\boldsymbol{p}_F,i\epsilon_n) \\ -f^*(\boldsymbol{r},-\boldsymbol{p}_F,i\epsilon_n) & g^*(\boldsymbol{r},-\boldsymbol{p}_F,i\epsilon_n) \end{pmatrix}, \quad (2)$$

depends on spatial coordinate $\boldsymbol{r}$, crystal momentum $\boldsymbol{p}_F = \hbar\boldsymbol{k}_F$ and energy ϵ_n, and must satisfy the normalization condition $\hat{g}^2 = \hat{1}$. g and f are normal and anomalous quasiclassical Green's function propagators and ϵ_n are fermionic Matsubara frequencies. Incorporated in this formalism is the self-consistent calculation of pair potential $\Delta(\boldsymbol{r})$ and $\boldsymbol{A}(\boldsymbol{r})$, which is essential for

the description of a superconductor hosting vortices as these two fields are dependent on each other. Although this approach is numerically much more feasible than direct diagonalization of a BdG Hamiltonian when treating inhomegeneties, it is not necessary to solve the problem completely self-consistently in this work. The reason for that is, that our experimental results clearly show, that even though the sub-gap states in the vortex display highly anisotropic behaviour, the recovery of $\Delta(\boldsymbol{r})$ is isotropic in the plane around the vortex core. The local pair potential is assumed to have s-wave symmetry and was therefore modeled by

$$\Delta(\boldsymbol{r}, \boldsymbol{p}_F) = \Delta(\boldsymbol{r}) = \left(\Delta_0 \tanh\frac{r}{\xi} + \Theta(z) W \tanh\frac{z}{a}\right)\left(\frac{x+iy}{r}\right)^m, \tag{3}$$

with Θ being a Heaviside step function, Δ_0 the maximum gap size, ξ the coherence length, a the lattice constant, m the winding number of the vortex and W the work function. The second term ensures, that quasiparticles travelling to the surface are decaying into the vacuum with the right damping factor. Since an isotropic Δ implies an isotropic in-plane current density, the magnetic field profile around the vortex was described by a vector potential of the form (*67*)

$$\boldsymbol{A} = A(r,z)\hat{\boldsymbol{e}}_\varphi\,, \tag{4}$$

$$A(r,z) = \frac{m\Phi_0}{2\pi\lambda^2}\int_0^\infty \mathrm{d}k \frac{J_1(kr)}{k^2+\lambda^{-2}} S(k,z)\,, \tag{5}$$

$$S(k,z) = \begin{cases} \frac{\kappa}{k+\kappa}\mathrm{e}^{-kz} & z > 0 \\ 1 - \frac{k}{k+\kappa}\mathrm{e}^{\kappa z} & z \leq 0\,, \end{cases} \tag{6}$$

that is cylinder symmetric in the bulk and deviates from the bulk value near and above the surface. Here, $\kappa = \sqrt{k^2+\lambda^{-2}}$, λ is the magnetic penetration depth which was chosen to be $\lambda = \xi/\sqrt{2}$ and $J_1(x)$ is a Bessel function of first order.

Nils Schopohl and Kazumi Maki (*68*) could show that the Eilenberger equations can always be solved along a characteristic line and that the solution is universal for each point along this line. The line simply has to be parallel to the Fermi velocity vector $\boldsymbol{v}_F$. Points on this line are

then characterized by the variable X and two *impact parameters* Y and Z.

$$\boldsymbol{r}(X) = X\hat{\boldsymbol{u}} + Y\hat{\boldsymbol{v}} + Z\hat{\boldsymbol{w}}, \tag{7}$$

$$= x\hat{\boldsymbol{x}} + y\hat{\boldsymbol{y}} + z\hat{\boldsymbol{z}} \tag{8}$$

Transformation from the mobile frame of reference $(\hat{\boldsymbol{u}}, \hat{\boldsymbol{v}}, \hat{\boldsymbol{w}})$, where $\hat{\boldsymbol{u}} \parallel \boldsymbol{v}_F$, to the fixed coordinate system $(\hat{\boldsymbol{x}}, \hat{\boldsymbol{y}}, \hat{\boldsymbol{z}})$ is done by chaining rotation matrices using Euler angles:

$$\begin{pmatrix} v_x \\ v_y \\ v_z \end{pmatrix} = R_{zyz} \begin{pmatrix} v_X \\ v_Y \\ v_Z \end{pmatrix} \tag{9}$$

$$\begin{aligned} R_{zyz} &= R_z(\eta) R_y(\chi - \pi/2) R_z(\psi) \\ &= \begin{pmatrix} \cos\eta & -\sin\eta & 0 \\ \sin\eta & \cos\eta & 0 \\ 0 & 0 & 1 \end{pmatrix} \begin{pmatrix} \sin\chi & 0 & \cos\chi \\ 0 & 1 & 0 \\ -\cos\chi & 0 & \sin\chi \end{pmatrix} \begin{pmatrix} \cos\psi & -\sin\psi & 0 \\ \sin\psi & \cos\psi & 0 \\ 0 & 0 & 1 \end{pmatrix}. \end{aligned} \tag{10}$$

In order to align the axis $\hat{\boldsymbol{u}}$ of the mobile frame with the axis $\hat{\boldsymbol{x}}$ of the static coordinate system, no rotation about $\hat{\boldsymbol{w}}$ is needed and thus $\psi = 0$. The other two rotational angles are defined by the components of the Fermi velocity vector as follows:

$$\eta = \arccos \frac{v_z}{|\boldsymbol{v}|}\,, \tag{11}$$

$$\chi = \begin{cases} \arctan \frac{v_y}{v_x}\,, & v_x > 0 \\ \arctan \frac{v_y}{v_x} + \pi\,, & v_x < 0\,. \\ \operatorname{sgn}(v_y)\frac{\pi}{2}\,, & v_x = 0 \end{cases} \tag{12}$$

Using this transformation, the vector potential and gap parameter can be expressed in the variables of the mobile frame: $A(\boldsymbol{r}(X, Y, Z))$ and $\Delta(\boldsymbol{r}(X, Y, Z))$. On a characteristic line defined by the impact parameters Y_p and Z_p, the functions from Eq. (1) become (*57*)

$$\Delta(X) = \Delta(\boldsymbol{r}(X, Y_p, Z_p))\,, \tag{13}$$

$$i\tilde{\epsilon}_n(X) = i\epsilon_n + \boldsymbol{v}_F e\boldsymbol{A}(\boldsymbol{r}(X, Y_p, Z_p))\,, \tag{14}$$

$$\hat{g}(X) = \hat{g}(\boldsymbol{r}(X, Y_p, Z_p), \boldsymbol{p}_F, i\epsilon_n). \tag{15}$$

The parametrisation of the Eilenberger equations on this 1D line is called the *Riccati parametrisation*. Eq. (1) formally reduces to the solution of two initial value problems, where the differential equations are scalar and of first order. The Eilenberger propagator is parametrised in terms of two scalar complex functions $a(X)$ and $b(X)$:

$$\hat{g}(X) = \frac{1}{1 + a(X)b(X)} \begin{pmatrix} 1 - a(X)b(X) & 2ia(X) \\ -2ib(X) & -1 + a(X)b(X) \end{pmatrix}. \tag{16}$$

The differential equations that need to be numerically solved for $a(X)$ and $b(X)$ are (*68*)

$$\hbar v_F a'(X) + [2\tilde{\epsilon}_n + \Delta^\dagger(X)a(X)]a(X) - \Delta(X) = 0\,, \tag{17}$$

$$\hbar v_F b'(X) - [2\tilde{\epsilon}_n + \Delta(X)b(X)]b(X) + \Delta^\dagger(X) = 0 \tag{18}$$

with boundary conditions

$$a(-\infty) = \frac{\Delta(-\infty)}{\epsilon_n + \sqrt{\epsilon_n^2 + |\Delta(-\infty)|^2}}\,, \tag{19}$$

$$b(+\infty) = \frac{\Delta^\dagger(+\infty)}{\epsilon_n + \sqrt{\epsilon_n^2 + |\Delta(+\infty)|^2}}. \tag{20}$$

In order to solve them at a certain energy $E < \Delta_0$, the analytical continuation $i\epsilon_n \to E + i0^+$ was used. Finally, the local density of states at $\boldsymbol{r}$ was obtained from the real part of $g(\boldsymbol{r}(X))$, i.e.

$$\mathcal{N}(\boldsymbol{p}_F) = \mathcal{N}_0(\boldsymbol{p}_F)\mathrm{Re}\left(\frac{1 - a(X)b(X)}{1 + a(X)b(X)}\right). \tag{21}$$

In order to obtain the total local density of states one still needs an integration over the Fermi surface to calculate all trajectories that are present due to the various Fermi velocity vectors that exist, plus an integration over the impact factors Y in order to account for trajectories that do not traverse the vortex centre. An integration over Z is redundant since the solution in the bulk is the same for every Z. Near the surface this is strictly not the case anymore but the effect is small and with a large enough variety of Fermi velocity vectors (which we have) these trajectories are not missed.

Influence of vector potential : The inclusion of a non-zero vector potential $\boldsymbol{A}$ in the calculations increases the average time needed to solve the Eilenberger equations because the Matsubara frequencies in Eq. (14) gain a position dependent term that requires a coordinate transformation between the two reference frames. Therefore, the simulations shown in the main text are performed without vector potential. It is already visible from Eq. (14) that in a calculation where $\boldsymbol{A}$ and Δ are not solved self-consistently, $\boldsymbol{A}$ only enters the equation as an effective energy term. With a vector potential in the azimuthal direction like in Eq. (5) the scalar product with the Fermi velocity is only expected to yield a substantial contribution for large impact parameters (for $Y_p = 0$, $\boldsymbol{v}_F$ is perpendicular to $\boldsymbol{A}$). That means trajectories with increasing impact parameter have large LDOS already for smaller distances than in the field free case. The splitting star arms in the LDOS maps should be squeezed to smaller distances from the core. In fact, this is what is seen in the calculations with vector potential at higher energies, as shown in Fig. S1. This proves that even though there are quantitative differences to the case without vector potential, in the general characteristics, the LDOS patterns remain unchanged.

Supplementary Note 1: Anomalous vortices in varying magnetic fields

The direction and magnitude of spatial displacement between the sets of CdGM state branches from the two superconducting bands found in anomalous vortices could not be tied to any crystal direction and appears to be almost random. By slowly varying the static magnetic field, the displacement can be modified, as shown in the dI/dU maps of the same anomalous vortex in Fig. S2 at different fields. We do, however, see an additional blue stripe in the zero-bias dI/dU maps of anomalous vortices that appears on the same side of the star pattern as the ring state. For clarity, the ring state is enclosed by a white-dashed circle and the borders of the mentioned stipe are highlighted by white-dashed contour lines in Fig. S2. These stripes might hint at the

direction in which the magnetic field lines are tilted beneath the surface.

As Fig. S3 demonstrates, a vortex core can be moved by a slow change in magnetic field and the sets of CdGM states from the two superconducting bands are displaced independently. Consequently, we are able to transform an anomalous vortex back to a normal vortex proving that the two types are not inherently different and that the anomalous type may well be explained by flux line tilting.

Supplementary Note 2: Giant vortex

In a single case, a giant vortex containing $m > 10$ flux quanta was found near a large sputtering defect. The zero-bias dI/dU map of this vortex, shown in Fig. S4A, features more than 10 arms of CdGM states stretching in each $\langle 2\bar{1}\bar{1}\rangle$ direction, which again split into pairs at larger bias (Fig. S4B). Unfortunately this particular giant vortex was partly outside the scan frame of the fine motion piezo tube, which prevented us from a definite determination of m.

Supplementary Note 3: Particle-hole and time-reversal symmetry

We find that the differential conductance maps show no qualitative change (compared to the figures in the main text) when the electric field in the tunnelling junction is reversed (inverse bias voltage sign $U \leftrightarrow -U$) or the magnetic field direction is reversed ($B\hat{\boldsymbol{e}}_z \leftrightarrow -B\hat{\boldsymbol{e}}_z$). As a point of proof, Fig. S5A and B show a normal vortex stabilized at $-14\,\mathrm{mT}$ (after saturation at $-85\,\mathrm{mT}$) for $U \geq 0\,\mathrm{mV}$ and Fig. S5C and D show an anomalous vortex stabilized at $18\,\mathrm{mT}$ for $U \leq 0\,\mathrm{mV}$. The invariance of the LDOS maps under reversal of the electric field demonstrates the particle-hole symmetry of the CdGM states and justifies their treatment as excitations of a BCS ground state, i.e. describing their dynamics by a mean-field BdG Hamiltonian or Eilenberger's quasiclassical Green's function propagators. The invariance of the LDOS maps under reversal

of the magnetic field is the logical consequence of time-reversal symmetry of the CdGM states.

Supplementary Note 4: Vortex at $4.3\,\mathrm{K}$

At $4.3\,\mathrm{K}$ the stabilization of single vortices within the STM scan frame was substantially harder. Upon repeating the magnetic protocol in small steps seven times, we only found a vortex within our scan frame in a single case. The vortex signature is shown in Fig. S6. At $U = 1.8\,\mathrm{mV} \approx \Delta/e$ (A), it appears as a round depression in differential conductance that is slightly smaller than at base temperature which is to be expected due to the temperature dependence of the coherence length $\xi(T) = \xi_0\sqrt{1 - T/T_c}$. At zero bias voltage (B), the CdGM states are smeared out. However, a conductance maximum is still found in the centre of the vortex (C) indicating that it is a vortex with odd winding number and, considering its size and shape, most likely $m = 1$. A distinction between Δ_1 and Δ_2 is not possible anymore at this temperature due to the temperature broadening of the dI/dU spectra (C).

Supplementary Note 5: Self-consistent calculation of Δ

We followed Ref. (*19*) and solved the Eilenberger equations in 2D self-consistently for $T = 0.1/7.2\,T_c$ in the clean limit, i.e. we refined the pair potential using the self-consistency equation (*19, 65*):

$$\Delta(\boldsymbol{r}) = 2\pi T\Lambda \sum_{n=0}^{N_c} S_F^{-1} \oint_{\mathrm{FS}} f(i\epsilon_n, \boldsymbol{r}, \boldsymbol{v}_F(\boldsymbol{k}))\mathrm{d}^2k \tag{22}$$

with

$$\Lambda = \left[\log(T/T_c) + \sum_{n=0}^{N_c} \frac{1}{n + 1/2}\right]^{-1}. \tag{23}$$

S_F is the Fermi surface area, $\epsilon_n = 2(n+1)\pi T$ the fermionic Matsubara frequencies and Λ is the coupling constant. We choose N_c such that $\epsilon_{N_c} = 5\,T_c$. Setting the vector potential $\boldsymbol{A} = 0$ and assuming an isotropic $\boldsymbol{v}_F$ we reach convergence for Δ with an accuracy of $10^{-4}\,T_c$. The result

for $\Delta(r)$ is displayed in Fig. S7 along with the function $\Delta(r) = \Delta_0 \tanh r/\xi_0$. For a distance from the vortex centre of $r < 2\xi_0$ the recovery of $\Delta(r)$ deviates from the function $\Delta_0 \tanh r/\xi_0$. The slope of $\Delta(r)$ close to $r = 0$ is roughly four times as steep as expected from a simple tanh behaviour with universal ξ_0. This leads to a core size $\xi^{(c)} = \Delta(\infty) \left[\lim_{r\to 0} \frac{\mathrm{d}\Delta(\mathrm{r})}{\mathrm{d}r}\right]^{-1}$ that is roughly $\xi_0/4$.

Supporting Data 1: Absence of ordered vortex pattern

We provide supporting data that clearly shows that our bulk Pb single crystal does not host an Abrikosov lattice (or any other kind of ordered pattern) of vortices, but that vortices coexist with large superconducting and normal domains in the intermediate state. In addition, we provide topographic images that show the atomically flat Pb surface below these vortices with no indication for flux pinning by defects.

Fig. S8 shows three connected areas for which we show the topographic scan image in A and the corresponding dI/dU map recorded at $U_t = 1.4\,\mathrm{mV}$ in B. The topographic image shows a typical Pb surface after our cleaning procedure. Here, we see six atomically flat and clean terraces (a seventh terrace begins just at the left edge of the leftmost scan frame) without larger defects at the surface. In the corresponding dI/dU map we see two vortices (round blue/purple objects marked by arrows), one larger normal conducting domain (purple area marked as “NC”) and a large superconducting domain (green/red area). The vortices obviously do not form an ordered pattern but coexist with large normal and superconducting domains in the intermediate state.

Supporting Data 2: dI/dU data for Fig. 3 and 4

We provide supporting data that shows the differential conductance (dI/dU) spectra from which Fig. 3 and Fig. 4J-K of the main text were derived. In Fig. 3 and Fig. 4J-K of the main text the

second derivative of dI/dU with respect to the bias voltage is shown. This method enhances visibility of peaks in the spectra and is commonly used for photoemission data. We now also show dI/dU as a function of bias voltage and the radial distance to the vortex centre as a 3D surface plot in Fig. S9 for the four different angles ($0°$, $20°$, $40°$, $60°$) which were defined in the main text and Fig. 3. The same we do for the two plots of Fig. 4J-K of the main text and show these 3D surface plots in Fig. S10. Since it is hard to follow the CdGM states as well as the gaps in one view, we also provide these interactive 3D surface plots in .html format in **3D-Data S1** to **S6**.

Table S1: Measurement parameters. I_t denotes the feedback condition of the tunnelling current at the bias voltage U_t. U_{ac}^{PK} is the peak AC voltage amplitude added via the Lock-in amplifier. In multi-pass dI/dU maps z_{off} is the distance the tip is brought closer to the surface compared to the feedback condition. B is the magnetic flux density in vacuum and T is the temperature.

Fig.		I_t(nA)	U_t(mV)	U_{ac}^{PK}(μV)	z_{off}(pm)	B(mT)	T(mK)
1	A	0.2	1	-	-	-	< 45
	B	0.05	1.3	50	-	23	< 45
	C	1	3	20	-	0	39
	D	2	3	20	-	23	48
2	A-G	0.5	1.4	50	20	0	< 45
	H	0.5	1.4	50	-	0	< 45
3		0.5	3	20	-	0	< 45
4	A-G	1	1.4	50	20	19	< 45
	H	1	1.4	50	-	19	< 45
	I	1	3	20	-	19	40
	J	1	1.5	10	-	19	40
	K	1	2	20	-	19	40
5	A-B	0.5	1.4	50	20	0	< 45
	C	0.5	1.4	50	-	0	< 45
	D-E	1	1.4	50	20	33	< 45
	F	1	1.4	50	-	33	< 45
	G	1	3	20	-	0	< 45
	H	1	3	20	-	33	< 45
S2	A-D	1	1.4	50	-	IND[a]	< 45
S3	A-B	0.2	1.4	50	-	IND	< 45
	C-D	1	1.4	20	-	IND	< 45
S4	A-B	1	3	50	20	0	< 45
S5	A-B	0.5	1.4	50	-	IND	36
	C-D	1	1.4	20	-	IND	42
S6	A	0.1	1.8	50	-	0	4300
	B	0.1	1.8	50	20	0	4300
	C	0.1	6	100	-	0	4300

[a] This parameter is indicated in the sub-figure itself.

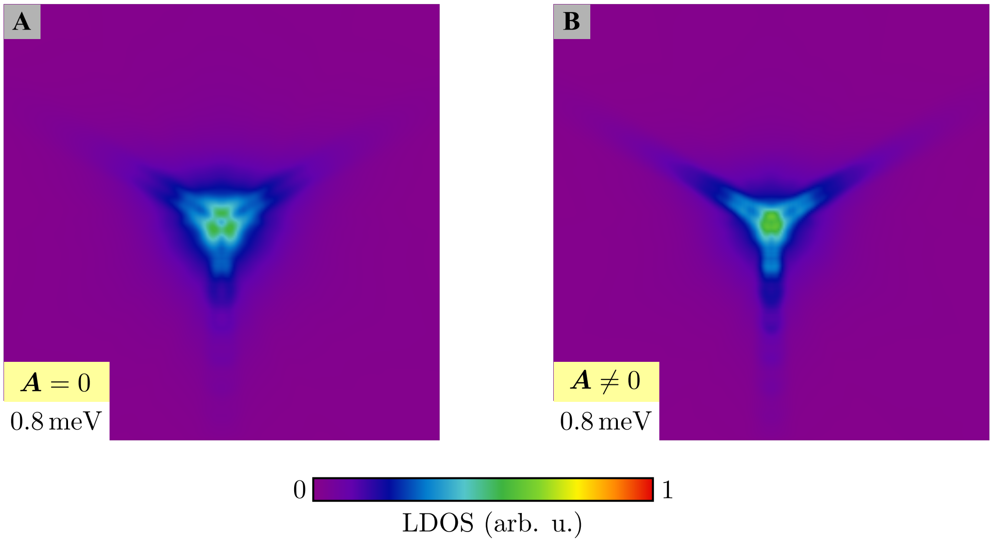

Figure S1: Influence of Vector Potential: The LDOS obtained from solutions of the 3D Eilenberger equations for a single-flux-quantum vortex at energy $eU = 0.8\,\mathrm{meV}$ without (**A**) and with vector potential (**B**), as formulated in Eq. (5), show only quantitative differences. With non-zero vector field, the star arms are still split, yet the CdGM states at this energy are squeezed into a smaller area around the vortex core.

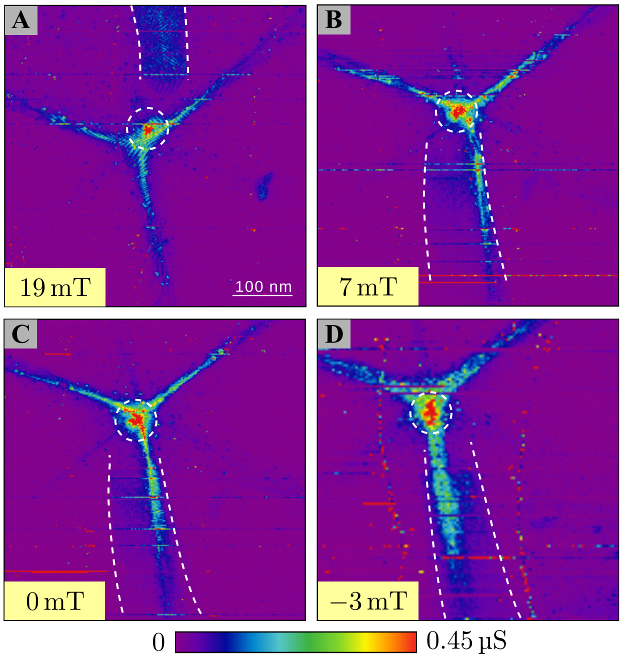

Figure S2: Manipulation of anomalous vortex pattern. Zero bias dI/dU maps of an anomalous vortex at different magnetic fields. The relative positional shift between the star centre and ring centre (red) is manifold and not tied to crystal directions. The position of the ring centre is marked by a white-dashed circle. The bright stripe mentioned in the main text is marked at its borders by white dashed lines.

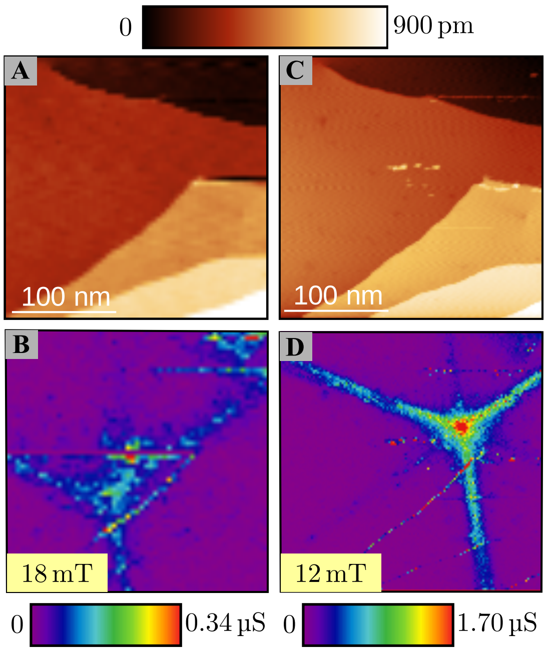

Figure S3: Manipulation from anomalous to normal vortex. (**A**,**C**) Topographic images of the corresponding quasiparticle patterns in (**B**,**D**) showing the same location on the surface. (**B**,**D**) Zero bias dI/dU map of the anomalous vortex before (**B**) and after the magnetic field ramp (**D**). After the decrease of magnetic field, the anomalous vortex in (**B**) moved and transformed into a normal type (**D**). In the process, both sets of CdGM states moved.

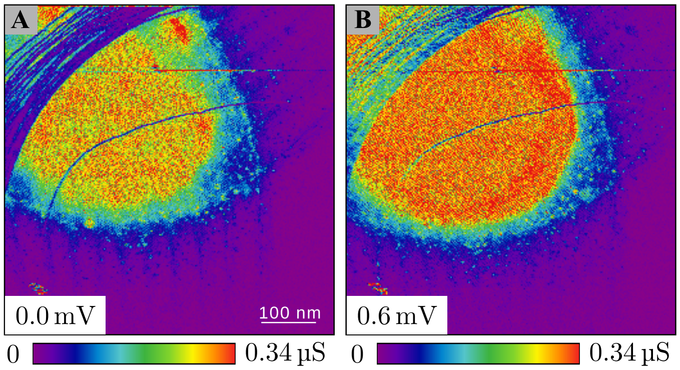

Figure S4: Giant vortex. dI/dU maps of a giant vortex containing $m > 10$ flux quanta. It shows more than 10 arms at zero bias (**A**) that individually split in two at sub-gap energies away from the Fermi level (**B**). This vortex is located at the edge of a large sputtering defect.

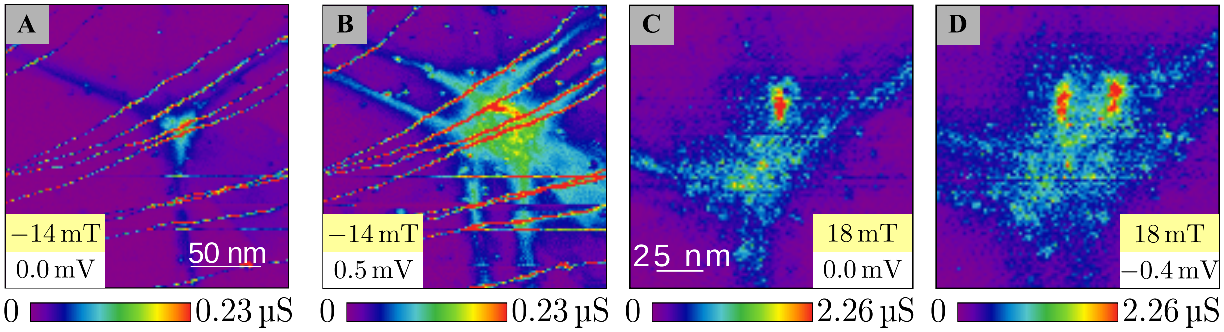

Figure S5: Reversal of magnetic and electric field. Applying the magnetic field or electric field in the opposite direction has no effect on the LDOS pattern inside the vortex. (**A**,**B**) $\mathrm{d}I/\mathrm{d}U$ maps of a normal single-flux-quantum vortex in reversed magnetic field. (**C**,**D**) $\mathrm{d}I/\mathrm{d}U$ maps of an anomalous vortex showing that reversing the sample bias yields indentical LDOS patterns.

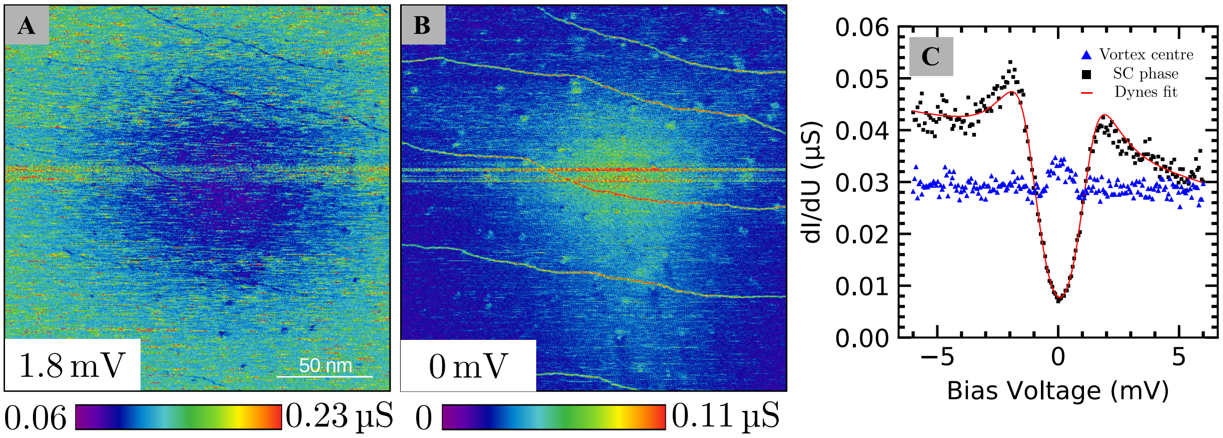

Figure S6: Vortex at higher temperature. (**A**,**B**) dI/dU maps of a vortex at $T = 4.3\,\mathrm{K}$ and $B = 0\,\mathrm{mT}$. (**C**) Bias spectroscopies far away from the vortex (black squares) and in the centre of the vortex (blue triangles) at $4.3\,\mathrm{K}$. The red line shows a fit of Dynes form to the differential conductivity in the superconducting phase with a gap size of $\Delta = 1.24\,\mathrm{meV}$.

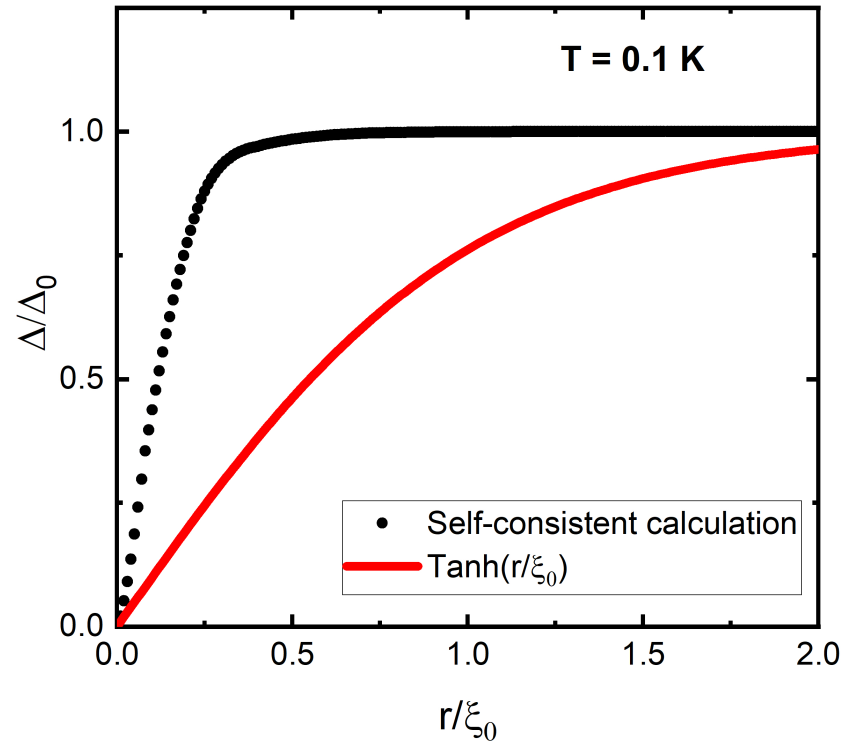

Figure S7: Kramer-Pesch effect: The self-consistent calculation of the pair potential Δ for a vortex with isotropic Fermi velocity at $T = 0.1/7.2\,T_c$ exhibits a shrinking of its core size according to the Kramer-Pesch effect, i.e. a steeper recovery of Δ close to the vortex centre that does not follow $\tanh r/\xi_0$.

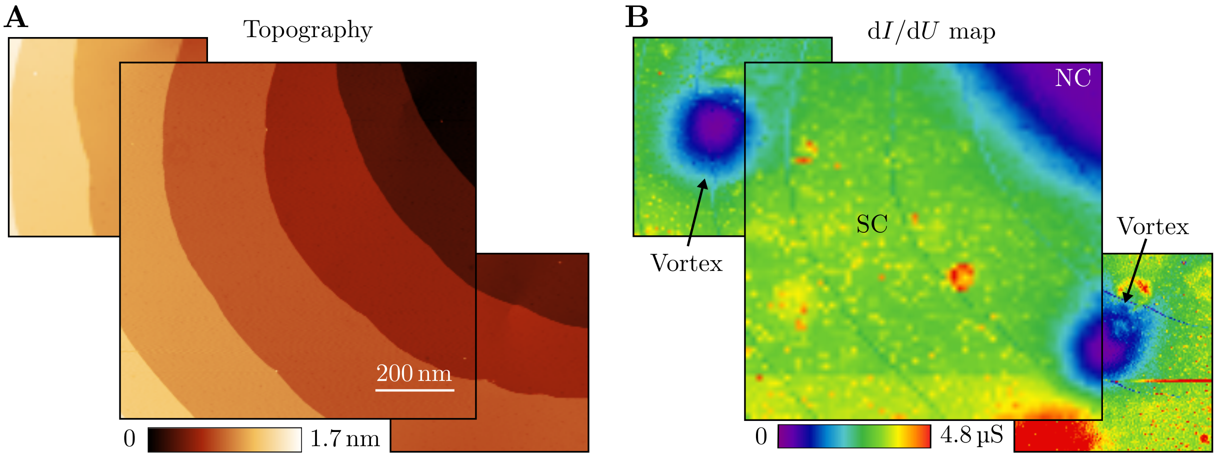

Figure S8: Absence of ordered vortex pattern: (**A**) Topographic image of three connected areas showing six atomically flat terraces. (**B**) dI/dU maps at $U_t = 1.4\,\mathrm{mV}$ and $B = 22\,\mathrm{mT}$ corresponding to the areas in (**A**) which show 2 vortices (round blue/purple objects marked by arrows) next to a larger normal conducting domain (purple area marked as "NC"). The green/red areas in the map belong to the superconducting parts (marked as "SC").

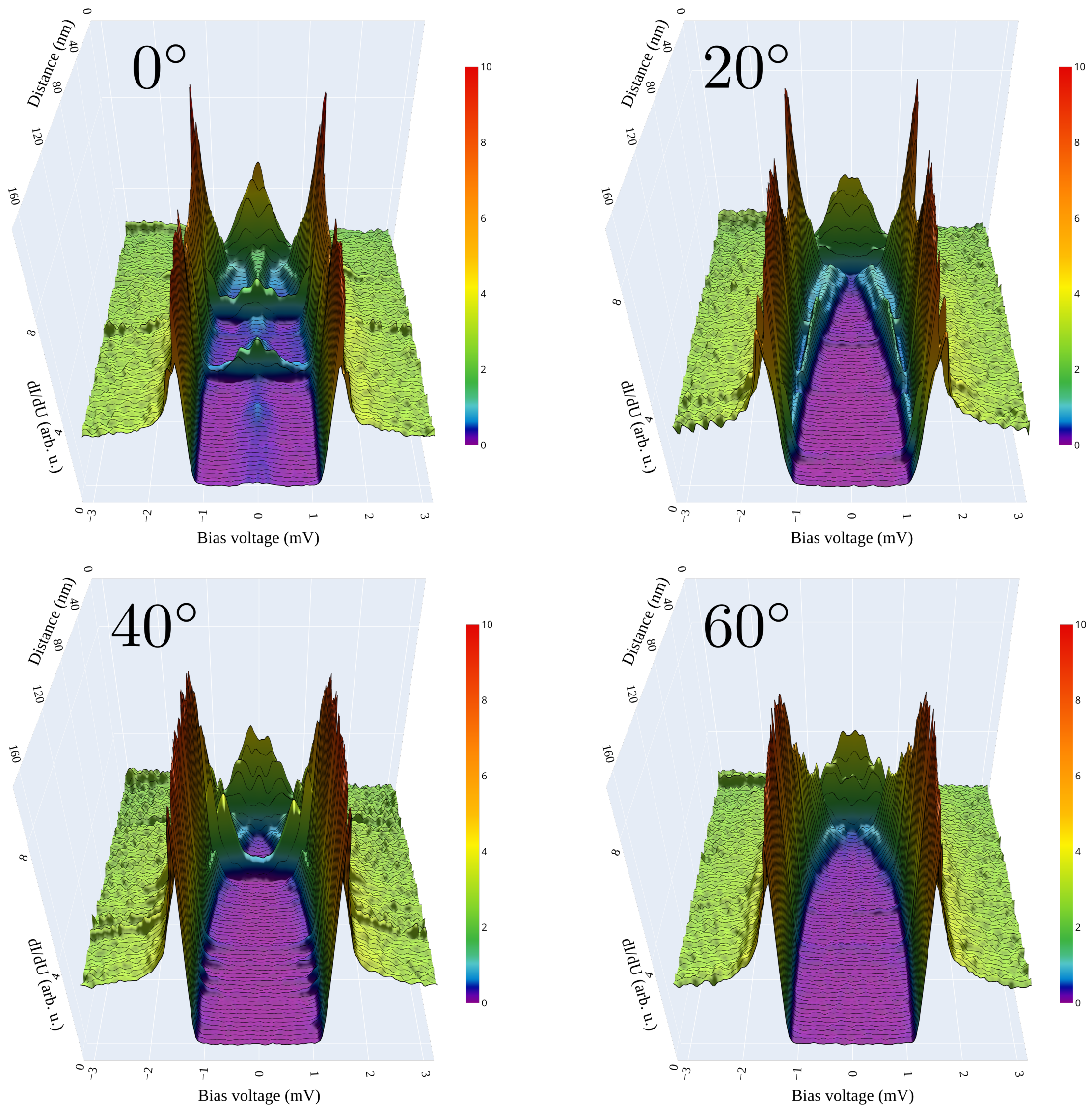

Figure S9: dI/dU spectra to Fig. 3: 3D surface plots of $\mathrm{d}I/\mathrm{d}U(r, U)$ for the four different angles as defined in Fig. 3 (0°, 20°, 40°, 60°). Bias voltage U is shown as x-coordinate, radial distance to the vortex centre r as y-coordinate and $\mathrm{d}I/\mathrm{d}U$ as color coded z-coordinate. The color encoding changes exponentially over the equally spaced values of the z-axis in order to emphasize small in-gap peaks corresponding to CdGM states and coherence peaks of larger intensity at the same time.

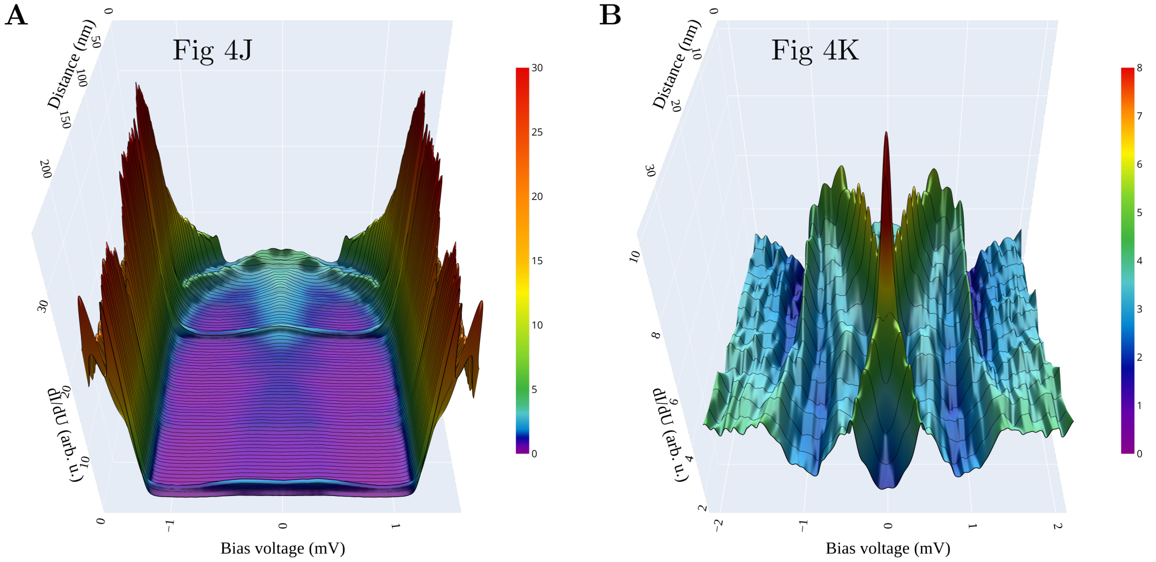

Figure S10: dI/dU spectra to Fig. 4: 3D surface plots of dI/d$U(r, U)$ for the two line profiles defined in Fig. 4I (differential form of spectra shown in Fig. 4J-K). Bias voltage U is shown as x-coordinate, radial distance to the vortex centre r as y-coordinate and dI/dU as color coded z-coordinate. For large range spectra the color encoding changes exponentially over the equally spaced values of the z-axis in order to emphasize small in-gap peaks corresponding to CdGM states and coherence peaks of larger intensity at the same time. (**A**) dI/d$U(r, U)$ spectra to Fig. 4J. (**B**) dI/d$U(r, U)$ spectra to Fig. 4K.

3D-Data S1: 3D data for Fig. 3. dI/dU as a function of bias voltage and the radial distance to the vortex centre as a 3D surface plot for $0°$ angle as defined in the main text in Fig. 3.

3D-Data S2: 3D data for Fig. 3. dI/dU as a function of bias voltage and the radial distance to the vortex centre as a 3D surface plot for $20°$ angle as defined in the main text in Fig. 3.

3D-Data S3: 3D data for Fig. 3. dI/dU as a function of bias voltage and the radial distance to the vortex centre as a 3D surface plot for $40°$ angle as defined in the main text in Fig. 3.

3D-Data S4: 3D data for Fig. 3. dI/dU as a function of bias voltage and the radial distance to the vortex centre as a 3D surface plot for $60°$ angle as defined in the main text in Fig. 3.

3D-Data S5: 3D data for Fig. 4J. dI/dU as a function of bias voltage and the radial distance to the vortex centre as a 3D surface plot for the profile in Fig. 4J of the main text.

3D-Data S4: 3D data for Fig. 4K. dI/dU as a function of bias voltage and the radial distance to the vortex centre as a 3D surface plot for the profile in Fig. 4K of the main text.

REFERENCES AND NOTES

1. V. L. Ginzburg, L. D. Landau, On the theory of superconductivity. *Zh. Eksp. Teor. Fiz.* **20**, 1064–1082 (1950).

2. L. D. Landau, Theory of supraconductivity. *Zh. Eksp. Teor. Fiz.* **7**, 371 (1937).

3. A. A. Abrikosov, On the magnetic properties of superconductors of the second group. *Sov. Phys. JETP* **5**, 1174 (1957).

4. C. N. Yang, Concept of off-diagonal long-range order and the quantum phases of liquid He and of superconductors. *Rev. Mod. Phys.* **34**, 694–704 (1962).

5. G. E. Volovik, Localized fermions on quantized vortices in superfluid ^{3}He-B. *J. Phys. Condens. Matter* **3**, 357–368 (1991).

6. E. B. Bogomol'nyi, A. I. Vainshtein, Stability of strings in gauge Abelian theory. *Sov. J. Nucl. Phys.* **23:5**, 7327784 (1976).

7. L. Jacobs, C. Rebbi, Interaction energy of superconducting vortices. *Phys. Rev. B* **19**, 4486–4494 (1979).

8. E. J. Weinberg, Multivortex solutions of the Ginzburg-Landau equations. *Phys. Rev. D* **19**, 3008–3012 (1979).

9. C. H. Taubes, ArbitraryN-vortex solutions to the first order Ginzburg-Landau equations. *Commun. Math. Phys.* **72**, 277–292 (1980).

10. A. V. Efanov, Exact *N*-vortex solutions to the Ginzburg-Landau equations for $\kappa=1/\sqrt{2}$. *Phys. Rev. B* **56**, 7839–7842 (1997).

11. I. Luk'yanchuk, Theory of superconductors with κ close to $1/\sqrt{2}$. *Phys. Rev. B* **63**, 174504 (2001).

12. V. Moshchalkov, M. Menghini, T. Nishio, Q. H. Chen, A. V. Silhanek, V. H. Dao, L. F. Chibotaru, N. D. Zhigadlo, J. Karpinski, Type-1.5 superconductivity. *Phys. Rev. Lett.* **102**, 117001 (2009).

13. E. Babaev, J. Carlström, J. Garaud, M. Silaev, J. M. Speight, Type-1.5 superconductivity in multiband systems: Magnetic response, broken symmetries and microscopic theory – A brief overview. *Phys. C Supercond.* **479**, 2–14 (2012).

14. R. Prozorov, R. W. Giannetta, A. A. Polyanskii, G. K. Perkins, Topological hysteresis in the intermediate state of type-I superconductors. *Phys. Rev. B* **72**, 212508 (2005).

15. G. D. Cody, R. E. Miller, Magnetic transitions of superconducting thin films and foils. I. Lead. *Phys. Rev.* **173**, 481–493 (1968).

16. G. J. Dolan, J. Silcox, Critical thicknesses in superconducting thin films. *Phys. Rev. Lett.* **30**, 603–606 (1973).

17. T. Cren, D. Fokin, F. Debontridder, V. Dubost, D. Roditchev, Ultimate vortex confinement studied by scanning tunneling spectroscopy. *Phys. Rev. Lett.* **102**, 127005 (2009).

18. A. Kamlapure, M. Simonato, E. Sierda, M. Steinbrecher, U. Kamber, E. J. Knol, P. Krogstrup, M. I. Katsnelson, M. Rösner, A. A. Khajetoorians, Tuning lower dimensional superconductivity with hybridization at a superconducting-semiconducting interface. *Nat. Commun.* **13**, 4452 (2022).

19. M. A. Silaev, V. A. Silaeva, Self-consistent electronic structure of multiquantum vortices in superconductors at $T \ll T_c$. *J. Phys. Condens. Matter* **25**, 225702 (2013).

20. T. Yamane, Y. Nagai, K. Tanaka, N. Hayashi, Impurity scattering effect on the zero-energy peak of the local density of states in a multi-quantum vortex core. *Phys. C Supercond.* **494**, 128–130 (2013).

21. Y. Masaki, Y. Kato, Impurity effects on Caroli–de Gennes–Matricon mode in vortex core in superconductors. *J. Physical Soc. Japan* **85**, 014705 (2016).

22. T. Cren, L. Serrier-Garcia, F. Debontridder, D. Roditchev, Vortex fusion and giant vortex states in confined superconducting condensates. *Phys. Rev. Lett.* **107**, 097202 (2011).

23. C. K. Poole, H. A. Farach, R. J. Creswick, *Handbook of Superconductivity* (Academic Press, 2000).

24. T. Balashov, M. Meyer, W. Wulfhekel, A compact ultrahigh vacuum scanning tunneling microscope with dilution refrigeration. *Rev. Sci. Instrum.* **89**, 113707 (2018).

25. M. Ruby, B. W. Heinrich, J. I. Pascual, K. J. Franke, Experimental demonstration of a two-band superconducting state for lead using scanning tunneling spectroscopy. *Phys. Rev. Lett.* **114**, 157001 (2015).

26. T. G. Saunderson, J. F. Annett, B. Újfalussy, G. Csire, M. Gradhand, Gap anisotropy in multiband superconductors based on multiple scattering theory. *Phys. Rev. B* **101**, 064510 (2020).

27. G. Chanin, J. P. Torre, Critical-field curve of superconducting lead. *Phys. Rev. B* **5**, 4357–4364 (1972).

28. R. Prozorov, Equilibrium topology of the intermediate state in type-I superconductors of different shapes. *Phys. Rev. Lett.* **98**, 257001 (2007).

29. R. Prozorov, A. F. Fidler, J. R. Hoberg, P. C. Canfield, Suprafroth in type-I superconductors. *Nat. Phys.* **4**, 327–332 (2008).

30. R. Prozorov, J. R. Hoberg, Dynamic formation of metastable intermediate state patterns in type-I superconductors. *J. Phys. Conf. Ser.* **150**, 052217 (2009).

31. C. Caroli, P. G. De Gennes, J. Matricon, Bound Fermion states on a vortex line in a type II superconductor. *Phys. Lett.* **9**, 307–309 (1964).

32. F. Gygi, M. Schlüter, Self-consistent electronic structure of a vortex line in a type-II superconductor. *Phys. Rev. B* **43**, 7609–7621 (1991).

33. G. E. Volovik, Vortex core anomaly from the gapless fermions in the core. *JETP Lett.* **58**, 455 (1993).

34. G. E. Volovik, Topological superfluids. *J. Exp. Theor. Phys.* **129**, 618–641 (2019).

35. S. M. M. Virtanen, M. M. Salomaa, Multiquantum vortices in superconductors: Electronic and scanning tunneling microscopy spectra. *Phys. Rev. B* **60**, 14581–14584 (1999).

36. S. M. M. Virtanen, M. M. Salomaa, Microscopic structure of multiquantum vortices in superconductors. *Phys. B Condens. Matter* **284-288**, 741–742 (2000).

37. H. F. Hess, R. B. Robinson, R. C. Dynes, J. M. Valles, J. V. Waszczak, Scanning-tunneling-microscope observation of the abrikosov flux lattice and the density of states near and inside a fluxoid. *Phys. Rev. Lett.* **62**, 214–216 (1989).

38. S. Hasegawa, T. Matsuda, J. Endo, N. Osakabe, M. Igarashi, T. Kobayashi, M. Naito, A. Tonomura, R. Aoki, Magnetic-flux quanta in superconducting thin films observed by electron holography and digital phase analysis. *Phys. Rev. B* **43**, 7631–7650 (1991).

39. J. Ge, J. Gutierrez, J. Cuppens, V. V. Moshchalkov, Observation of single flux quantum vortices in the intermediate state of a type-I superconducting film. *Phys. Rev. B* **88**, 174503 (2013).

40. Y. Tanaka, A. Hasegawa, H. Takayanagi, Energy spectrum of the quasiparticle in a quantum dot formed by a superconducting pair potential under a magnetic field. *Solid State Commun.* **85**, 321–326 (1993).

41. K. Tanaka, I. Robel, B. Jankó, Electronic structure of multiquantum giant vortex states in mesoscopic superconducting disks. *Proc. Natl. Acad. Sci. U.S.A.* **99**, 5233–5236 (2002).

42. L. D. Landau, The intermediate state of supraconductors. *Nature* **141**, 688 (1938).

43. L. D. Landau, On the theory of the intermediate state of superconductors. *J. Phys. USSR* **7**, 99 (1943).

44. S. Conti, F. Otto, S. Serfaty, Branched microstructures in the Ginzburg-Landau model of type-I superconductors. arXiv:1507.00836 (2015).

45. H. F. Hess, R. B. Robinson, J. V. Waszczak, Vortex-core structure observed with a scanning tunneling microscope. *Phys. Rev. Lett.* **64**, 2711–2714 (1990).

46. M. V. Berry, M. Wilkinson, Diabolical points in the spectra of triangles. *Proc. R. Soc. Lond. A Math. Phys. Sci.* **392**, 15–43 (1997).

47. A. Odobesko, F. Friedrich, S.-B. Zhang, S. Haldar, S. Heinze, B. Trauzettel, M. Bode, Anisotropic vortices on superconducting Nb(110). *Phys. Rev. B* **102**, 174502 (2020).

48. H. Kim, Y. Nagai, L. Rózsa, D. Schreyer, R. Wiesendanger, Anisotropic non-split zero-energy vortex bound states in a conventional superconductor. *Appl. Phys. Rev.* **8**, 031417 (2021).

49. L. Kramer, W. Pesch, Core structure and low-energy spectrum of isolated vortex lines in clean superconductors at $T \ll T_c$. *Z. Phys.* **269**, 59–64 (1974).

50. M. Ichioka, V. G. Kogan, J. Schmalian, Locking of length scales in two-band superconductors. *Phys. Rev. B* **95**, 064512 (2017).

51. M. Silaev, E. Babaev, Microscopic theory of type-1.5 superconductivity in multiband systems. *Phys. Rev. B* **84**, 094515 (2011).

52. V. G. Kogan, J. Schmalian, Ginzburg-Landau theory of two-band superconductors: Absence of type-1.5 superconductivity. *Phys. Rev. B* **83**, 054515 (2011).

53. A. J. Leggett, Number-phase fluctuations in two-band superconductors. *Prog. Theor. Phys.* **36**, 901–930 (1966).

54. V. H. Dao, L. F. Chibotaru, T. Nishio, V. V. Moshchalkov, Giant vortices, rings of vortices, and reentrant behavior in type-1.5 superconductors. *Phys. Rev. B* **83**, 020503 (2011).

55. A. V. Samokhvalov, V. D. Plastovets, A. S. Mel'nikov, Topological transitions in electronic spectra: Crossover between Abrikosov and Josephson vortices. *Phys. Rev. B* **102**, 174501 (2020).

56. S. Rex, R. Willa, A topological flux trap: Majorana bound states at screw dislocations. *New J. Phys.* **24**, 053057 (2022).

57. N. Schopohl, Transformation of the Eilenberger equations of superconductivity to a scalar Riccati equation. arXiv:cond-mat/9804064 (1998).

58. C. Elsässer, N. Takeuchi, K. M. Ho, C. T. Chan, P. Braun, M. Fähnle, Relativistic effects on ground state properties of 4D and 5D transition metals. *J. Phys. Condens. Matter* **2**, 4371–4394 (1990).

59. B. Meyer, M. Fähnle, Ab initio calculation of the formation energy and the formation volume of monovacancies in Mo. *Phys. Rev. B* **56**, 13595–13598 (1997).

60. D. Vanderbilt, Optimally smooth norm-conserving pseudopotentials. *Phys. Rev. B* **32**, 8412–8415 (1985).

61. L. Kleinman, Relativistic norm-conserving pseudopotential. *Phys. Rev. B* **21**, 2630–2631 (1980).

62. R. Heid, K.-P. Bohnen, I. Y. Sklyadneva, E. V. Chulkov, Effect of spin-orbit coupling on the electron-phonon interaction of the superconductors Pb and Tl. *Phys. Rev. B* **81**, 174527 (2010).

63. J. P. Perdew, Y. Wang, Accurate and simple analytic representation of the electron-gas correlation energy. *Phys. Rev. B* **45**, 13244–13249 (1992).

64. T.-S. Choy, J. Naset, J. Chen, S. Hershfield, C. Stanton, A database of Fermi surface in virtual reality modeling language (VRML). *Bull. Am. Phys. Soc.* **45**, L36–L42 (2000).

65. G. Eilenberger, Transformation of Gorkov's equation for type II superconductors into transport-like equations. *Z. Phys.* **214**, 195–213 (1968).

66. A. I. Larkin, Y. N. Ovchinnikov, Quasiclassical method in the theory of superconductivity. *Sov. Phys. JETP* **28**, 1200 (1969).

67. G. Carneiro, E. H. Brandt, Vortex lines in films: Fields and interactions. *Phys. Rev. B* **61**, 6370–6376 (2000).

68. N. Schopohl, K. Maki, Quasiparticle spectrum around a vortex line in a d-wave superconductor. *Phys. Rev. B* **52**, 490–493 (1995).
